# Male pseudohermaphroditism in a complex malformed calf born with an acardius amorphus cotwin—a case report

**DOI:** 10.1186/s12917-023-03639-8

**Published:** 2023-07-18

**Authors:** Hiromi Kusaka, Makoto Sugiyama, Satoshi Kameshima, Takehiko Kakizaki, Yasunori Suzuki, Ryo Ando, Hiroshi Miura, Motohiro Kikuchi, Hiroaki Kawaguchi, Minoru Sakaguchi

**Affiliations:** 1 Laboratory of Theriogenology, School of Veterinary Medicine, Kitasato University, Towada, Aomori, 034-8628 Japan; 2 Laboratory of Veterinary Anatomy, School of Veterinary Medicine, Kitasato University, Towada, Aomori, 034-8628 Japan; 3 Laboratory of Small Animal Internal Medicine, School of Veterinary Medicine, Kitasato University, Towada, Aomori, 034-8628 Japan; 4 Laboratory of Veterinary Radiology and Radiation Biology, School of Veterinary Medicine, Kitasato University, Towada, Aomori, 034-8628 Japan; 5 Laboratory of Animal Hygiene, School of Veterinary Medicine, Kitasato University, Towada, Aomori, 034-8628 Japan; 6 Laboratory of Veterinary Pathology, School of Veterinary Medicine, Kitasato University, Towada, Aomori, 034-8628 Japan; 7 Veterinary Clinical Education Promotion Office, School of Veterinary Medicine, Kitasato University, Towada, Aomori, 034-8628 Japan

**Keywords:** Acardius amorphus cotwin, Immunohistochemistry staining, *In situ* hybridization, Japanese Black calf, Male pseudohermaphroditism

## Abstract

**Background:**

Male pseudohermaphroditism is a developmental anomaly wherein animals are genetically and gonadally male, but their internal and/or external genitalia resemble those of females. In cattle, pseudohermaphroditism is often accompanied by multiple severe malformations. To the best of our knowledge, this is the first report of male pseudohermaphroditism in a complex malformed calf born with an acardius amorphous cotwin.

**Case presentation:**

This report describes the case of a three-day-old, male anurous Japanese Black calf born with an acardius amorphous cotwin, complete absence of the tail, agenesis of the anus, separate scrota, and umbilical hernia. Transthoracic echocardiography and computed tomography revealed serious malformations in the skeletal system and the circulatory, digestive, urinary, and genital organs. Necropsy revealed rectal atresia, immature testes, epididymis, and penis, but no male accessory gonads. Histological analyses revealed vaginal- and uterine-like tissues adjacent to or fused to the rectum. Fluorescence *in situ* hybridization detected X and Y chromosomes, and some cells presented two X-probe signals in the same nucleus.

**Conclusions:**

In contrast to the male genitalia, the female genitalia derived from the Müllerian ducts were difficult to detect by necropsy in the presented case. Many similar cases may be overlooked in clinical practice.

**Supplementary Information:**

The online version contains supplementary material available at 10.1186/s12917-023-03639-8.

## Background

The term ‘intersex’ refers to animals of equivocal sex in which physical characteristics, including the external genitalia, are inconclusive. Intrinsic hermaphroditism refers to individuals that have both ovaries and testes or ovotestis with a mixture of both reproductive tissues. Pseudohermaphroditism is an abnormality of phenotypic sex, which refers to the presence of gonads of a single sex and the alteration of one or more of the other criteria for sex identification [1].

Male pseudohermaphrodites are genetically and gonadally male, but their internal and/or external genitalia resemble those of the females. Male pseudohermaphrodites are more common than female or true hermaphrodites, which is likely because more genes (steroidogenic enzymes, 5α; β-reductase, and androgen receptors) are required to initiate male development, providing greater opportunity for genetic defects. There have been few case reports of male pseudohermaphrodites in cattle [2, 3].

In cattle, multiple severe malformations have been reported in previous cases of pseudohermaphroditism, particularly in females. In these cases, there was an absence of the anus and tail, a deficit of urethral and vaginal perineal openings, and ambiguous external genitalia [4–6]. Pathological examination also confirmed rectal atresia, internal genitourinary, and skeletal anomalies.

To the best of our knowledge, there have been no cases of male pseudohermaphroditism in a complex malformed calf born with an acardius amorphus cotwin. This report describes a case of male pseudohermaphroditism in a complex malformed calf born with an acardius amorphus cotwin.

## Case presentation

### Case history

A male Japanese Black calf was born with an acardius amorphus cotwin (φ9.5 cm) on a commercial farm. These calves originated from the transplantation of two frozen embryos that were produced by multiple ovulation embryo transfer (MOET). The calf was presented at the Kitasato University Large Animal Medical Center because of atresia ani three days after calving, while the acardius amorphus was lost during transportation. The calf showed a suckling reflex and was able to drink milk through the bottle. On admission, physical examination of the calf revealed it was anurous, agenesis of the anus, separate scrota, and umbilical hernia. The calf was unable to stand up from birth owing to skeletal deformity of the back. Cardiac auscultation revealed a pansystolic heart murmur.

### Clinical examination

The calf underwent a complete blood count (CBC), biochemical examination of blood, and ultrasonography of the chest and abdomen. The CBC values were within the reference intervals, but the cell findings from blood smears showed an increased ratio of juvenile neutrophils. Biochemical examination revealed hyperkalemia and hypernatremia (7.61 and 141.1 mEq/L, respectively), and that blood urea nitrogen, creatinine, total bilirubin, and γ-glutamyl transpeptidase were elevated to 44.3 mg/dL, 12.1 mg/dL, 3.2 mg/dL, and 67.2 IU/L, respectively. Transthoracic echocardiography revealed a left-to-right shunting atrial septal defect with right atrial dilatation and a subaortic ventricular septal defect.

The calf was then scanned using computed tomography (CT; Aquilion 16, Toshiba Medical Systems, Tokyo, Japan) under general anesthesia to better understand the spatial relationship of the organs in the pelvic cavity. CT revealed partial agenesis of the lumbar and sacral vertebrae and caudal vertebral agenesis (Supplementary Fig. 1). The rectum was located inside the pelvic cavity and ended completely before the ischial tuber. Additionally, a uterine-like structure existed below the rectum, while the region from the uterine-like structure to the urethra was unclear. Because the calf died during the CT examination, a necropsy was performed.

### Necropsy

**Fig. 1:**
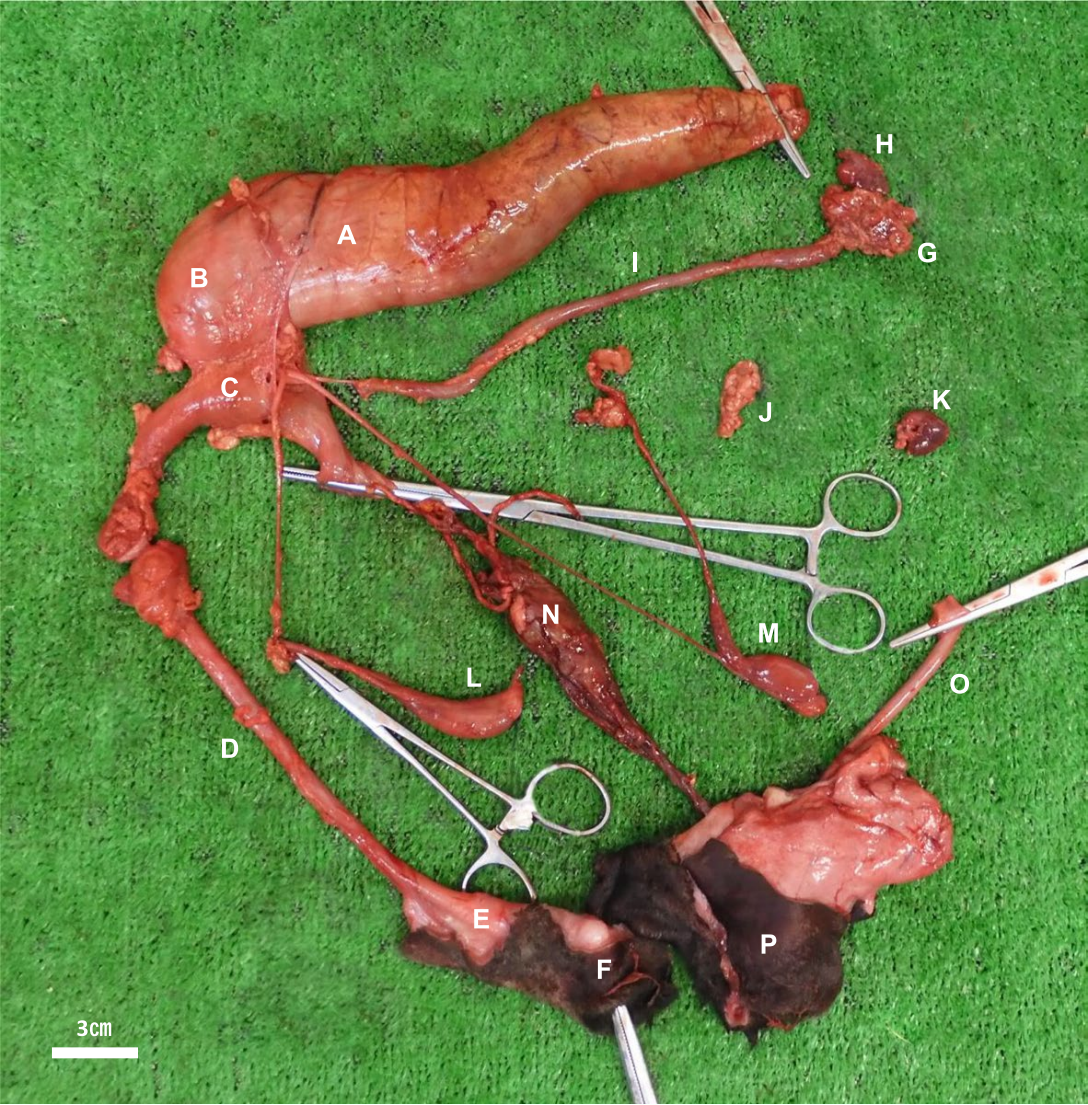
Gross appearance of the rectum and urogenital organs of the calf. (**A**) rectum, (**B**) vaginal-like structure, (**C**) uterine-like structure, (**D**) urethra, (**E**) penis, (**F**) external urethral orifice, (**G**) right kidney, (**H**) right adrenal gland (**I**) right urinary duct, (**J**) left kidney, (**K**) left adrenal gland, (**L**,** M**) right and left testis, (**N**) urachus, (**O**) umbilical vein, and (**P**) navel. Scale bar = 3 cm

Table 1 summarizes the malformations of the skeletal system and organs. Postmortem gross evaluation of the digestive tract confirmed rectal atresia. The rectum and colon were filled with gas and feces. The rectal end was closed, and the distance between the ends of the atretic rectum and the absent anal opening was approximately 6.0 cm (Fig. 1A). A vaginal-like structure was observed at the end of the rectum (Fig. 1B). The urinary system also abnormally developed. Instead of the bladder, a uterine-like structure with an internal cavity was observed at the bottom of the rectum (Fig. 1C). The end of the structure was closed and adhered strongly to the rectum, forming a small fistula. Additionally, the kidneys were notably small (Fig. 1G, J). The left renal artery and ureter were absent, the right renal artery and ureter were hypoplastic, and the end of the right ureter was blind and fused to the tubular structure under the rectum. Gross examination of the gonads and internal genitals revealed that the testes, epididymis, and deferent duct were largely normal (Fig. 1L, M); however, there were no accessory gonads. The deferent duct was connected to a uterine-like structure, while the details of connecting part were not clear. External genitals such as the penis and urethra were normal but did not clearly present a connection to the internal genitourinary system (Fig. 1D–F).

**Table 1:** Post-mortem gross malformations

Items	Details
Skeletal system	・Uneven number of left and right ribs (14 versus 13 ribs, respectively).
	・1 subtrahend of the lumbar spine.
	・Fusion of the transverse process of the 5th lumbar vertebra and the sacral wing of the 1st sacral vertebra.
	・Defect from 2nd sacral vertebra to the caudal vertebra with spinal cord defect after cauda equina.
	・Defect in the caudal vertebra.
	・Deformation of the pelvis (insufficiency of ischial arch dilatation).
Circulatory organs	・Ventricular septal defect (Diameter: 1.7 cm).
	・Patent foramen ovale (Diameter: 1.5 cm).
	・Patent ductus arteriosus (Diameter: 0.5 cm).
Digestive organs	・Absence of anus
	・Rectal atresia
Urinary organs	・Hypoplasia of the left and right kidneys.
	・Aplasia in the left renal artery and ureter.
	・Hypoplasia of right renal artery and ureter.
Genital organs	・Defects in the male accessory gonads.
	・Indistinct opening of vas deferens

### Tissue histology

During necropsy, each organ of the calf was removed for evaluation and subsequently fixed in 10% neutral buffered formalin for histopathological studies using routine hematoxylin & eosin (HE) and immunohistochemistry (IHC) staining. Fixed tissue samples were embedded in paraffin and sectioned at a thickness of 4 μm. For IHC staining, the deparaffinized sections were incubated with 0.3% H_2_O_2_ in methanol and blocked with 3% bovine serum. Following blocking, the sections were incubated at 4 ºC at 17 h with the primary antibodies, anti-cytokeratin 8 antibody (CK8) (5 μg/ml Cusabio, Houston, TX) and anti-cytokeratin 14 antibodies (CK14) (0.5 μg/ml; BioLegend, San Diego, CA). The sections were subsequently incubated with the secondary horseradish peroxidase (HRP)-conjugated antibody, anti-rabbit IgG antibody (Dilute kit 1:4; Nichirei, Tokyo, Japan), for 60 min at 24 ºC. The antigen/antibody complexes were visualized with 3,3’-diaminobenzidine tetrahydrochloride staining using the Histofine Simple Stain MAX-PO kit (Nichirei, Tokyo, Japan), as previously described [7].

**Fig. 2:**
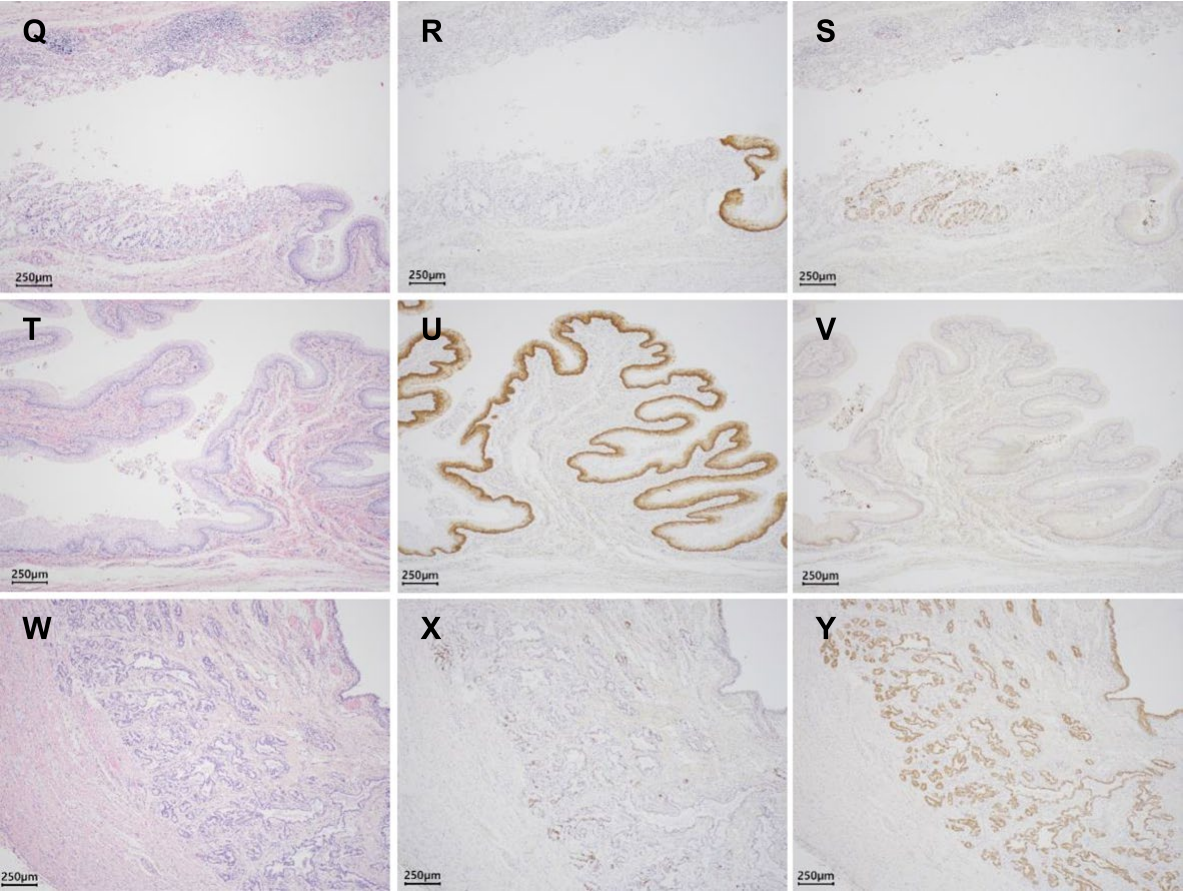
Hematoxylin and eosin (H & E) and immunohistochemial (IHC) stains of female reproductive tissues. (**Q**–**S**) Transition from rectum to vaginal-like tissue. (**T**–**V**) Vaginal-like tissue (stratified squamous epithelium). (**W**–**Y**) Uterine-like tissue (endometrial and uterine gland-like tissues). (**Q**, **T**, **W**) H & E stain. (**R**, **U**, **X**) CK14 IHC. (**S**, **V**, **Y**) CK8 IHC. Scale bar = 250 µm

**Fig. 3:**
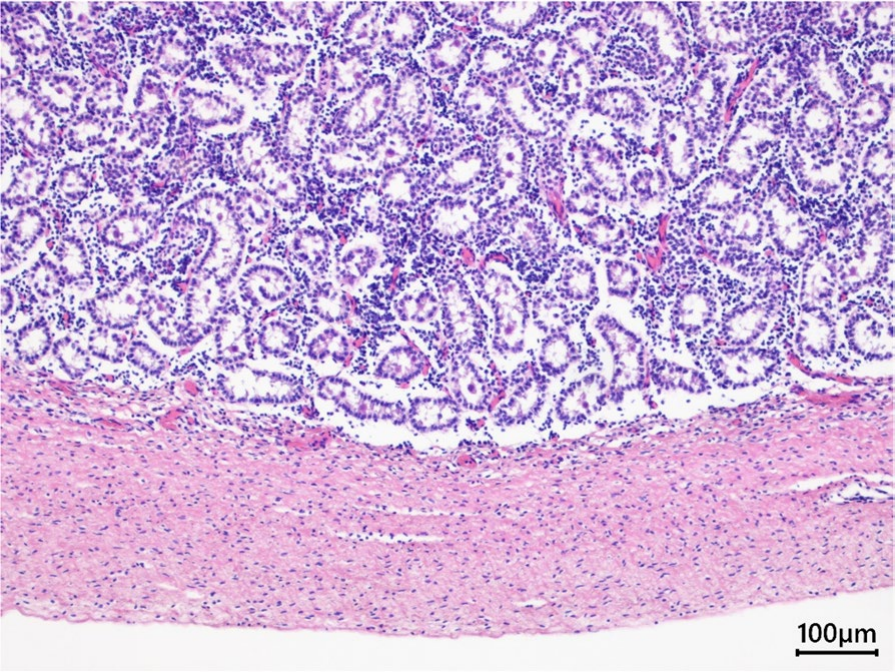
Hematoxylin and eosin (H&E) stain of immature testis tissues of the male tissue. Scale bar = 100 µm

The end of the rectum (closed part) consisted of vaginal-like tissue (stratified squamous epithelium) that migrated from the gastrointestinal mucosal epithelium (Figs. 1B and  2Q, T). The uterine-like tissue (endometrium and uterine gland-like tissues) was adjacent to the rectum and opened with a small fistula (Fig. 1C, 2W). Male tissues such as immature testis, epididymis, and penis were also histologically present (Fig. 3). IHC examination of the gastrointestinal mucosal epithelium in the rectum was positive for CK8, whereas no CK14 was detected (Fig. 2R, S). The stratified squamous epithelium in vaginal-like tissue revealed strong positive detection of CK14 (Fig. 2R, U), and only a slight detection (mainly basement membrane) of CK8 (Fig. 2S, V). The endometrium and uterine gland-like tissues in uterine-like tissue revealed positive detection of CK8, while showing a slight detection (mainly basement membrane) of CK14 (Fig. 2X, Y).

### X and Y chromosome detection by PCR and *in situ* hybridization

PCR and *in situ* hybridization (ISH) were performed to identify the X and Y chromosomes. Fresh blood was used for PCR using genomic DNA. In addition, frozen blood samples from healthy male and female Japanese Black calves were used as positive controls.

PCR-based chromosomal sex determination was conducted as previously described to determine the presence of the bovine *SRY* and amelogenin (*AMEL*) loci [8]. The PCR primers used to amplify the 480 bp *SRY* region were 5’-TCGTGAACGAAGACGAAAGGTGGC-3’ upstream and 5’-GCACAAGAAAGTCCAGGCTCTAAGC-3’ downstream. The primers used to amplify a 280 bp fragment from the X-chromosome *AMEL* sequence and a 217 bp Y-chromosome *AMEL* sequence were 5’-CAGCCAAACCT-CCCTCTGC-3’ upstream and 5’-CCCGCTTGGTCTTGTCTGTTGC-3’ downstream. The KOD-FX neo (Toyobo, Osaka, Japan) polymerase was used. PCR amplification was performed for 36 cycles at 94 ºC for 10 s, 66 ºC for 30 s, and 68 ºC for 30 s. To sequence the PCR amplicons, the PCR products were purified using the QIAquick gel extraction kit (Qiagen GmbH) after agarose gel electrophoresis. Next, index-tagged libraries for each purified DNA sample were prepared using the Nextera XT DNA Library Preparation Kit (Illumina, Inc., San Diego, CA, USA) and sequenced using the Illumina iSeq system with 150-bp paired-end reads according to the manufacturer’s instructions. The reads obtained from iSeq were trimmed and filtered using the CLC Genomics Workbench 22.0 (Qiagen GmbH) set to a minimum length of 100 bp, and a quality score threshold of 30. These trimmed reads were assembled *de novo* with the “*De Novo Assembly*” mode and default settings.

ISH was performed to determine the X and Y chromosomes from a section of the vaginal-like structure. The X- and Y-probes used for ISH were biotin-labeled and fluorescein-5-isothiocyanate (FITC)-labeled kits (Chromosome Science Labo Inc. Sapporo, Japan). Male Japanese Black calf spleen tissue sections were used as positive controls. Probes were denatured at 85 ºC for 1 min and hybridized in a moist chamber at 37 ºC for 17 h. After hybridization and slide washing, the biotin-labeled probe was visualized using a red DyLight 549 fluorochrome conjugated to streptavidin (Vector Laboratories, Burlingame, CA, USA). The slides were counterstained with 4′,6-diamidino-2-phenylindole (DAPI) in an antifade mounting medium (Thermo Fisher Scientific, Waltham, MA, USA). The sections were observed using an LSM710 confocal microscope (Carl Zeiss, Oberkochen, Germany).

**Fig. 4:**
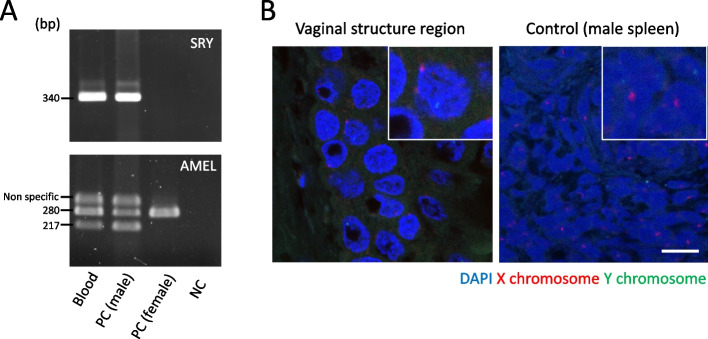
Chromosomal sex-determination. Expression of Y- and X-chromosome specific sequences determination by polymerase chain reaction (PCR) (**A**) and *in situ* hybridization (ISH) (**B**). (**A**) The *AMEL* and *SRY* loci detected by PCR in blood. In males, 340 bp of *AMEL* nonspecific sequence was amplified. PC; positive control, NC; negative control. Negative control used water as the template. (**B**) Fluorescence-ISH images. X-chromosomal probes labeled with biotination and detected by streptavidin DyLight 549 (red), Y-chromosomal probes were detected by fluorescein-5-isothiocyanate (FITC) (green). Nuclei were counterstained with 4′,6-diamidino-2-phenylindole (DAPI). The white boxed shows higher magnification from the figure. Scale bars = 10 µm

PCR analysis of genomic DNA from the blood detected Y chromosome-specific sequences at both the *SRY* and *AMEL* loci (Fig. 4A). This 340 bp amplified product was found to be a repeat sequence on the Y chromosome. In contrast, the two bands below the 340 bp band were amplicons of the amelogenin genes located on the X or Y chromosome. The 217 bp product was amplified from the *AMEL* loci on the Y chromosome (accession NM_174240.2., Y-linked amelogenin mRNA, position 389–605) and the 280 bp product from the *AMEL* loci on the X chromosome (Accession: NM_001014984.1, X-linked amelogenin mRNA, position 389–668), respectively. Fluorescence-ISH of vaginal-like tissue similarly detected X- and Y-chromosomes (Fig. 4B). X- and Y-hybridized probes were detected in colon-like and uterine-like areas (Supplementary Fig. 2), whereas in vagina-like tissue, two X-probed cells were observed in the same nucleus.

## Discussion and conclusions

Histopathological and IHC examinations revealed the presence of uterine/vaginal-like tissue in this male calf. The ISH test implied confusion of the cells with XY and XX chromosomes in the vaginal tissues. Because of the presence of Y-specific DNA sequences in the patient’s blood sample, this patient was diagnosed with male pseudohermaphroditism, and was genetically and gonadally male but with internal genitalia resembling those of a female. Furthermore, severe multiple malformations and/or hypoplasia were observed in this case. To the best of our knowledge, this is the first case of pseudohermaphroditism in a complex malformed calf born with an acardius amorphus cotwin.

In normal male individuals, the presence of anti-Müllerian hormones and androgens is necessary for the development of the internal reproductive organs. Anti-Müllerian hormone secreted by the Sertoli cells in the testis causes the Müllerian duct to regress, whereas androgen secreted by the Leydig cells differentiates the Wolffian duct into the epididymis, deferent duct, ejaculatory duct, and seminal gland [1]. This case was defined as genetically male because the patient possessed external male genitalia and Y-specific DNA sequences. In contrast, the patient had immature testes, abnormal development and location of Müllerian ducts, and the presence of uterine/vaginal-like tissues. Possible reasons for the persistence of Müllerian ducts include: 1) impaired production of anti-Müllerian hormones, a genital differentiation inducer secreted by Sertoli cells, or 2) impaired sensitivity due to abnormal receptors for anti-Müllerian hormones.

With ambiguous internal genitalia, this patient presented with multiple serious malformations in the skeletal system and circulatory, digestive, and urinary organs. In cattle, a series of malformations have often been reported in female pseudohermaphroditism [4–6]. Similar complex intersex and serious malformations have also been reported in one dizygotic twin lamb [9] and in a foal [10]. To the best of our knowledge, only one case has been described of male pseudohermaphroditism in complex malformations in a kitten [11]. Similar to previous reports, the presented case showed multiple severe malformations and/or hypoplasia of the genitourinary system and ambiguous internal genitalia. Moreover, organs of different embryological origins, gastrointestinal tract, and genital tract were continuously fused in the patient.

Several theories have been proposed regarding the pathogenesis of serious malformations affecting multiple organs around the urogenital tract. Escobar et al. (1987) proposed that a growth deficiency in the urorectal septum and improper induction of mesenchymal tissue in the presence of an intact cloacal membrane results in ambiguous genitalia [12]. Wheeler and Weaver (1997 or 2001) also hypothesized that partial development of the urorectal septum and incomplete breakdown of the cloacal membrane results in severe and multiple anomalies [9, 13]. They suggested that mechanical interference due to the persistence of the cloaca causes developmental abnormalities in internal genital organs. Additionally, primary polytopic developmental field defects cause a series of abnormalities, defined as urorectal septum malformation sequence (URSMS) in these human fetal disorders. Sonic hedgehog (*SHH*) and homeobox (*HOX*) genes are involved in mesodermal activity during blastogenesis [14]. Spontaneous or teratogenic mutations in these genes lead to Gli protein amplification abnormalities and deficient or altered gene expression that results in caudal mesodermal deficiency [14]. In the present case, no cloacae remained, which differs from general human URSMS. Simultaneously, the symptoms are also different from those of hermaphroditism, as dysplasia occurs in multiple organs in the lumbar and abdominal cavities. Unraveling the commonalities and differences among the cases accumulated thus far as well as the novelty of the present case will require advances in genetic approaches.

In most cases, genetic factors, environmental factors, or a combination of both may cause a series of malformations, but their etiology remains obscure [15]. Here, the afflicted patient was one of the dizygotic twins and the cotwin was an acardius amorphus fetus. Therefore, the fact that two calves with the same genetic background developed severe malformations at the same time suggests that the severe malformations in this calf may not only be familial but may also be the result of a strong chromosomal disorder (e.g., fertilized egg preservation status). Assisted reproduction technologies, such as MOET and in vitro fertilization-embryo transfer (IVF), are toxic to embryos as they are exposed to various external environments outside of the cow’s body. In humans, the incidence of congenital malformations after IVF has been reported [16], but not after MOET. In this case, the factors that cause complex malformations are very complicated. 

Regarding the condition of ambiguous internal genitalia, sex chromosome confusion could disrupt normal organogenesis during early development because this patient had cells with both XY and XX chromosomes. In cattle, heterosexual twins can exchange hematopoietic stem cells or white blood cells from the vascular anastomosis of the allantois. Freemartin is a classic example of a chimera in cytogenetics, containing two or more cell types that originate in separate individuals and are infertile due to anatomical defects in the female reproductive tract. Males born to cotwins with females may also be chimeric and have low reproductive efficiency [17]. Acardius amorphus in cattle often appears in heterosexual twins [17–19]. Although our study could not investigate the sex of the acardius amorphus, it is hypothesized that the presence of the acardius amorphus explained the status of the sex chromosome chimera in this patient.

In this case, chromosome testing, a standard genetic test, was not conducted as an antemortem diagnosis. Therefore, the influence of changes in the number or structure of the entire chromosome on the development of complex intersex and serious malformations in this patient is unclear. However, using ISH, we were able to report for the first time the occurrence of chromosomal chimerism in a case of male pseudohermaphroditism in complex malformations in cattle. Fluorescence-ISH is a test used to map genetic material in cells, allowing the visualization of specific genes or portions of genes. A combination of conventional methods and genetic information, including ISH, will provide important new insights into the disease in question.

In conclusion, this report describes the first confirmed case of male pseudohermaphroditism in a complex malformed calf born with an acardius amorphus cotwin, diagnosed by histopathological, IHC, and ISH examinations. We initially diagnosed the patient as a pseudohermaphrodite. However, in pseudohermaphrodites, the malformations are limited to the genital organs; in the present case, the malformations spread to multiple organs. This is probably also the case in many clinical fields, where there are discrepancies in diagnosis due to the lack of recognition of intersex conditions accompanied by a series of malformations. Accruing knowledge regarding male pseudohermaphroditism with complex malformations will contribute to elucidating the mechanism and etiology of its development in veterinary medicine.

## Supplementary information

**Additional file 1.** The original PCR analysis images for Fig. 4A.

**Additional file 2: Supplementary figure 1.**

**Additional file 3: Supplementary figure 2.**

## Data Availability

The datasets used and/or analyzed during the current study are available from the corresponding author upon reasonable request.

## Abbreviations

AMEL: Amelogenin
CBC: Complete blood count
CK14: Anti-cytokeratin 14 antibodies
CK8: Anti-cytokeratin 8 antibody
CT: Computed tomography
DAPI: 4′,6-Diamidino-2-phenylindole
FITC: Fluorescein-5-isothiocyanate
H&E: Hematoxylin & eosin
HOX: Homeobox
HRP: Horseradish peroxidase
IHC: Immunohistochemistry
ISH: *in situ* Hybridization
SHH: Sonic hedgehog
URSMS: Urorectal septum malformation sequence
