# Supplementary material for: Male pseudohermaphroditism in a complex malformed calf born with an acardius amorphus cotwin—a case report
Source: BMC Vet Res. 2023 Jul 18;19:86. doi: 10.1186/s12917-023-03639-8 (PMC10353092; doi:10.1186/s12917-023-03639-8)
Supplement: Supplementary file 1 — Additional file 1. The original PCR analysis images for Fig. 4A. [file 12917_2023_3639_MOESM1_ESM.pdf]

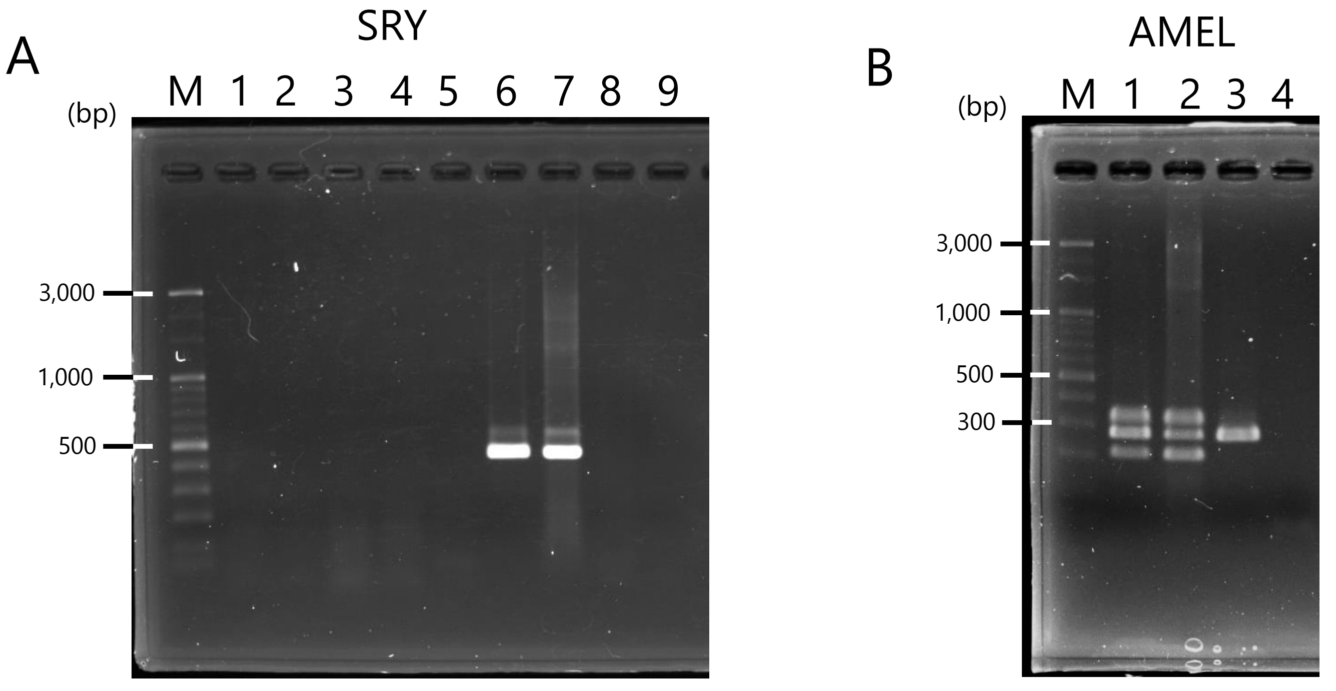

The original PCR analysis images for Fig. 4A. (A) The target SRY analyzed by PCR was amplified from DNA templates extracted from formalin-fixed patient tissues (Lane 1–5) and frozen blood (Lane 6). Lane 1: colon. Lane 2: vagina. Lane 3: uterus. Lane 4: liver. Lane 5: spleen. Lane 6: blood. Lane 7: blood as male positive control. Lane 8: blood as female positive control. Lane 9: water as negative control. (B) The target AMEL analyzed by PCR was amplified from DNA templates extracted from frozen blood (Lane 1). Lane 2: blood as male positive control. Lane 3: blood as female positive control. Lane 4: water as negative control. M, DNA marker (bp) (Loading Quick 100bp DNA ladder, Eurofins Scientific SE, Luxembourg).
