# Supplementary material for: Male pseudohermaphroditism in a complex malformed calf born with an acardius amorphus cotwin—a case report
Source: BMC Vet Res. 2023 Jul 18;19:86. doi: 10.1186/s12917-023-03639-8 (PMC10353092; doi:10.1186/s12917-023-03639-8)
Supplement: Supplementary file 2 — Additional file 2: Supplementary figure 1. [file 12917_2023_3639_MOESM2_ESM.pdf]

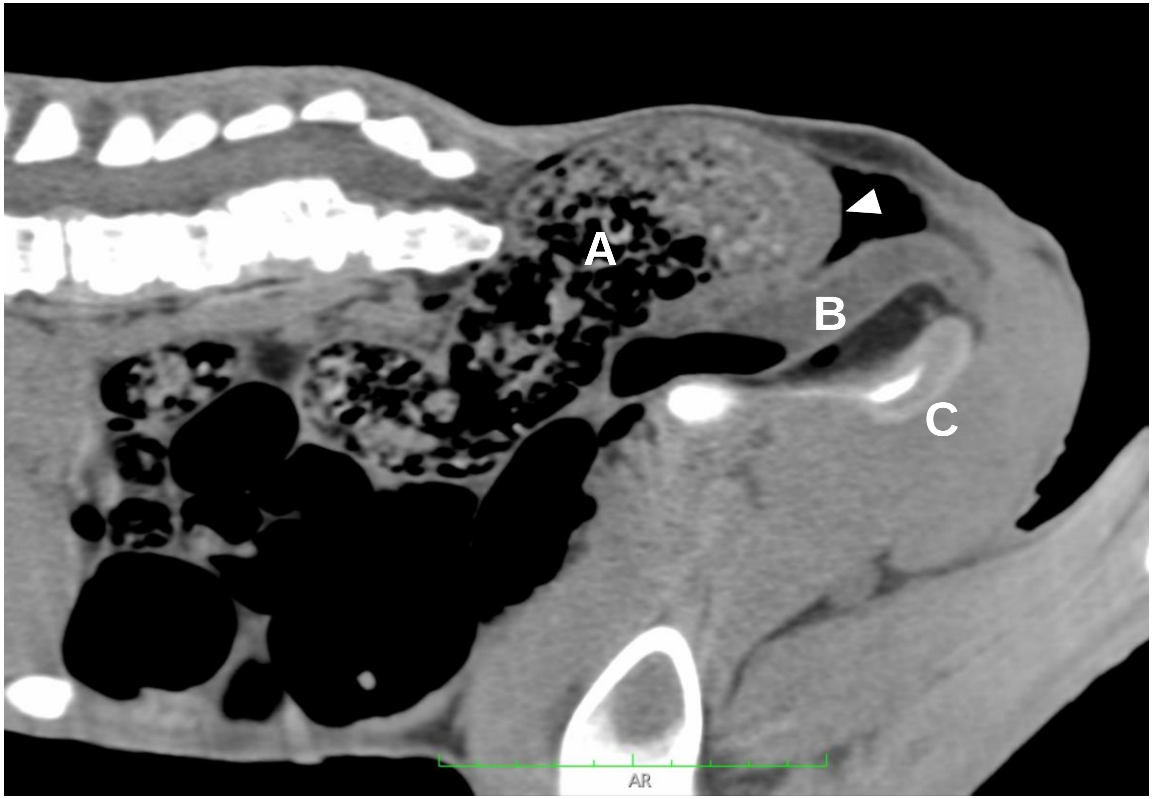

Supplementary figure 1. Longitudinal section computerized tomography (CT) scan from the lumber region to the hip.

(A) Rectum, the rectal end was closed (arrowhead), (B) uterine-like structure, (C) ischium bone. Scale bar = 1 cm.
