# Supplementary material for: Male pseudohermaphroditism in a complex malformed calf born with an acardius amorphus cotwin—a case report
Source: BMC Vet Res. 2023 Jul 18;19:86. doi: 10.1186/s12917-023-03639-8 (PMC10353092; doi:10.1186/s12917-023-03639-8)
Supplement: Supplementary file 3 — Additional file 3: Supplementary figure 2. [file 12917_2023_3639_MOESM3_ESM.pdf]

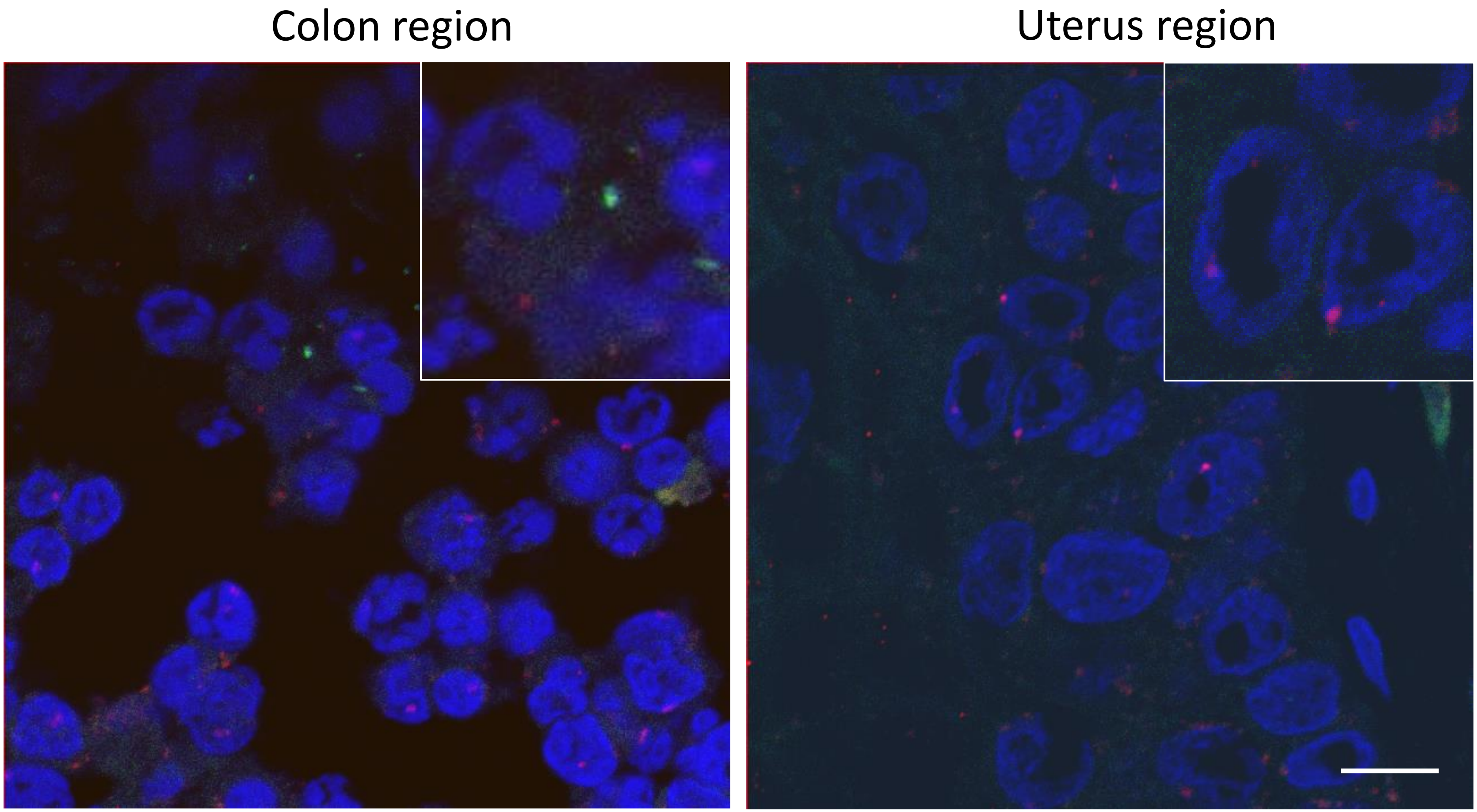

Supplementary figure 2. Representative fluorescence in situ hybridization (ISH) images of the colon and uterus regions.

X-chromosomal probes labeled with biotin and detected by streptavidin DyLight 549 (red), Y-chromosomal probes were detected by fluorescein-5-isothiocyanate (FITC) labeling (green). The nuclei were counterstained with 4′,6-diamidino-2-phenylindole (DAPI). The white box indicates higher magnification. Scale bar = 10 µm.
